# Practices of strength and conditioning coaches in professional sports: a systematic review

**DOI:** 10.5114/biolsport.2022.107480

**Published:** 2021-08-30

**Authors:** Anthony Weldon, Michael J. Duncan, Anthony Turner, Robert G. Lockie, Irineu Loturco

**Affiliations:** 1 Human Performance Laboratory, The Technological and Higher Education Institute of Hong Kong, Hong Kong; 2 Centre for Sport, Exercise and Life Sciences, Coventry University, Coventry, United Kingdom; 3 London Sport Institute, Middlesex University, London, United Kingdom; 4 Center for Sport Performance, Department of Kinesiology, California State University, Fullerton, Fullerton, CA, United States of America; 5 NAR – Nucleus of High Performance in Sport, São Paulo, Brazil; 6 Department of Human Movement Sciences, Federal University of São Paulo, São Paulo, Brazil; 7 University of South Wales, Pontypridd, Wales, United Kingdom

**Keywords:** Team Sports, Individual Sports, Performance Training, Coaching, Strength Training, Physical Training

## Abstract

The practices of strength and conditioning (S&C) coaches have been reported in various professional sports. This study aims to comprehensively assess this available evidence to help establish whether theoretical, practical, and evidence-based guidelines align with the practices employed by these experienced S&C coaches. Three databases were searched (PubMed, SPORTDiscus, and Cochrane) until November 2020. Studies surveying the practices of S&C coaches in professional sports using a survey design with common questions, written in English, and published in peer-reviewed journals were reviewed. Eight studies (*n* = 318 S&C coaches) were finally included. All studies adapted a similar survey, providing a strong basis for comparison between sports. Periodization strategies were widely used (89%), with training volume consistently reduced during the in-season period. Olympic weightlifting was commonly used across sports, except in baseball (29%). Plyometric exercises were predominantly prescribed for speed development (74%) and lower body power (68%), which were mostly programed as complex training (45%) and conducted all year round (52%). Flexibility exercises were mostly performed before practice (83%) for 6–10 min (40%). Physical tests were mainly conducted during the preseason period (66%), with body composition (86%) being the most used test. S&C coaches generally adhered to current guidelines and research in S&C concerning training prescription and physical testing. Whereas, intersport differences were also noted and further discussed. Results of this study can be used by S&C coaches to plan, implement, and review their professional practices. Furthermore, may inform the development of general and sport-specific guidelines, and future research in S&C.

## INTRODUCTION

Professional sports continue to evolve, and with an exponential increase in available funds, sports organizations are more commonly employing comprehensive multidisciplinary athlete support teams [1]. The responsibilities of these staff span across technical, medical, sport science, and physical training sectors [2]. One role that has become increasingly important, is the strength and conditioning (S&C) coach [3–4], who is interconnected with numerous aspects of athlete preparation [5]. Accordingly, S&C coaches are required to possess a broad understanding of other departments within the multidisciplinary team (e.g., sports scientists and head coaches) [5]. The main objectives of S&C coaches are to enhance the physical and sports performance of athletes while reducing the likelihood of injury [3]. Given the broad roles and responsibilities, and increasingly high expectations placed on S&C coaches, particularly at the elite level, it is important to explore their perspectives and practices [4].

Various studies have previously investigated the practices of S&C coaches in different professional sports, including American football [6], ice hockey [7], baseball [8], basketball [9], wrestling [10], rugby union [11], soccer [12], and cricket [13]. All the aforementioned studies adapted a survey originally used in American football [6], and although each study further examined alternative areas of S&C, the original content of the survey was consistently applied. Each survey addressed the practices of S&C coaches related to strength, power, speed, plyometric, and flexibility training, in addition to physical testing.

The benefits of the aforementioned training methods and use of physical testing to comprehensively develop athletes have been extensively researched and integrated into S&C guidelines [14]. Whereas little is known how S&C guidelines and research are used by S&C coaches in elite level sport. For example, it is recognized that general and sport-specific strength training is commonly integrated into S&C training programs using periodization strategies, with the aim of improving elite athletes’ sports performance and resilience to injury [15]. However, given the difficulties in conducting such research at the elite level and scarcity of available data [15–17], it is proposed that researchers may need to use alternative methods to explore and understand the use and efficacy of S&C training methods [15].

Therefore, the objective of this systematic review was to comprehensively assess the evidence from prior surveys investigating the practices of S&C coaches in different professional sports. This will help identify whether theoretical, practical, and evidence-based guidelines align with the practices employed by these experienced S&C coaches. In turn, providing a basis for further advancing S&C as a discipline through informing future directions for professional development and research on this topic.

## MATERIALS AND METHODS

**FIG. 1:**
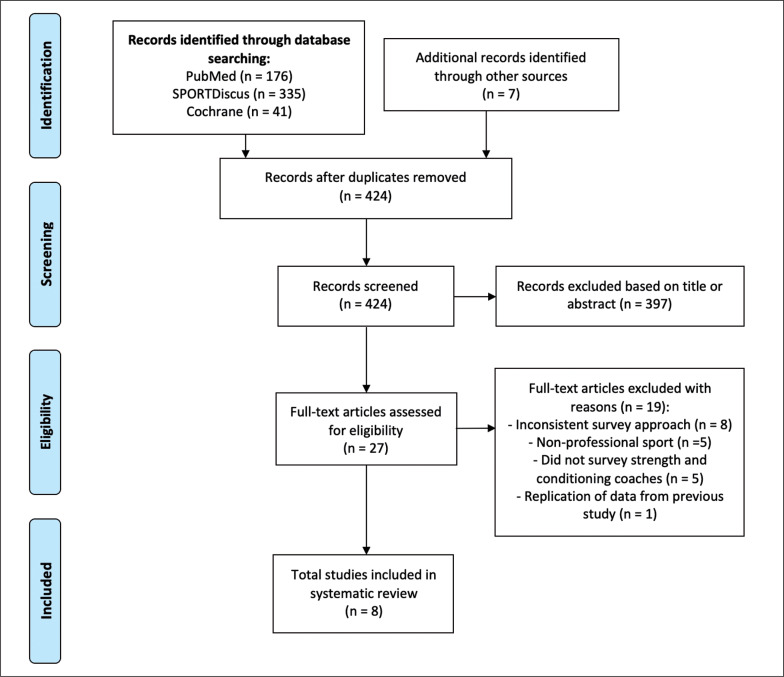
Preferred Reporting Items for Systemativ review and Meta-Analysis Protocols (PRISMA-P) flowchart illustrating the inclusion and exclusion criteria used in the systematic review.

This study was performed using the Preferred Reporting Items for Systematic Review and Meta-Analyses Protocol (PRISMA-P) [18], as presented in Figure 1.

### Eligibility Criteria

Studies were eligible if they met the following inclusion criteria: (a) written in English, (b) published in a peer-reviewed journal, (c) used a survey design with common questions, and (d) the survey’s purpose was to investigate the practices of S&C coaches in professional sports. The lead author (AW) performed a detailed investigation during the planning stage of the systematic review to ensure the selected criteria were relevant.

### Information Sources and Search Strategy

Searches for studies were conducted by the lead author (AW) from the 1^st^ November 2020 until the 12^th^ November 2020, using three electronic databases considered suitable for systematic reviews (PubMed, SPORTDiscus, and Cochrane) [19]. Search terms were modified to the settings and limitations of the respective databases, with the following keywords combined using Boolean operators: Practices of strength and conditioning coaches in* OR strength and conditioning practices in* OR (elite sport* or professional sport* or national sport* or international sport*). The reference lists of selected studies were searched for additional suitable studies.

### Quality Check

An adapted Critical Appraisal Skills Programme (CASP) [20] checklist for qualitative research, was deemed most appropriate to assess the reporting quality of included studies in this systematic review. The CASP checklist consists of three sections, with two initial screening questions and eight further questions exploring the validity and applicability of results to the relevant population. Each question is graded as either: yes, can’t tell, or no. Section A evaluates the validity of the results of each study, including the following questions: 1) Was there a clear statement of the aims of the research?; 2) Was a qualitative or mixed quantitative and qualitative methodology appropriate?; 3) Was the research design appropriate to address the aims of the research?; 4) Was the recruitment strategy appropriate to the aims of the research?; 5) Was the data collected in a way that addressed the research issue?; and 6) Has the relationship between researcher and participants been adequately considered?. Section B evaluates the quality of results, and includes the following questions: 7) Have ethical issues been taken into consideration?; 8) Was the data analysis sufficiently rigorous?; and 9) Is there a clear statement of findings?. Section C evaluates whether the results will help locally and includes the following question: 10) Is the research valuable?.

Any disagreements between the decisions of reviewers (AW and MJD) were discussed, and if unresolved settled by a third reviewer (AT).

### Data Collection Process

The characteristics of all studies included in the review were manually extracted into a customized Excel workbook (Microsoft Excel 2019, Microsoft Corporation, Redmond, Washington, USA). The data extracted included: (1) study identification information, (2) study appraisal rating, (3) sample size, (4) sport, (5) league, (6) level of sport, (7) country, (8) frequency and duration of off-season and inseason strength and power training sessions, (9) most important exercises, (10) use of Olympic weightlifting, (11) use of periodization strategies, (12) methods for determining set loads, (13) speed development exercises (e.g., types of exercise), (14) plyometric exercises (e.g., types of exercises and purpose), (15) flexibility exercises (e.g., types of exercise and when performed), (16) physical testing (e.g., types of tests and when implemented), and (17) mean values for each included variable. If data were missing for an included variable, all authors reviewed the manuscript and confirmed whether not attainable (n/a) should be used.

## RESULTS

### Study Selection

Overall 559 studies were identified using the search strategy outlined in Figure 1. Following the removal of duplicates and articles discarded based on the review of titles and abstracts by two reviewers (AW and MJD), 27 articles were determined relevant for further analysis. Thereafter, two reviewers (AW and MJD) read the full texts of selected studies, compared results, and reached a consensus on which studies to be included in the systematic review. Finally, 8 studies were selected, checked for quality, and agreed by all reviewers to be used for data synthesis. General information regarding the eligible studies is presented in Table 1.

**TABLE 1:** Characteristics of reviewed studies on the practices of strength and conditioning coaches in professional sports.

Study	Sample Size	Response Rate	Sport	League	Level of sport	Country
Ebben et al. [6]	26	87%	AF	National Football League	Professional Team	USA
Ebben et al. [7]	23	76.6%	Ice Hockey	National Hockey League	Professional Team	USA
Ebben et al. [8]	21	70%	Baseball	Major League Baseball	Professional Team	USA
Simenz et al. [9]	20	68.9%	Basketball	National Basketball Association	Professional Team	USA
Far Saeed et al. [10]	100	88.5%	Wrestling	Iran Wrestling League	Professional Team	Iran
Jones et al. [11]	43	83%	Rugby Union	Various Leagues	Professional Team	Global
Weldon et al. [12]	52	ND	Soccer	Various Leagues	Professional and International Teams	Global
Weldon et al. [13]	33	ND	Cricket	Various Leagues	Professional and International Teams	Global

AF: American Football; ND: Not determined; USA: United States of America.

### Quality Check

The results from the CASP checklist are presented in Table 2.

**TABLE 2:** Critical appraisal using The Critical Appraisal Skills Programme (CASP) checklist for qualitative research.

Study	Q1	Q2	Q3	Q4	Q5	Q6	Q7	Q8	Q9	Q10
Ebben et al. [6]	Y	Y	Y	Y	Y	CT	CT	Y	Y	Y
Ebben et al. [7]	Y	Y	Y	Y	Y	CT	Y	Y	Y	Y
Ebben et al. [8]	Y	Y	Y	Y	Y	CT	CT	Y	Y	Y
Simenz et al. [9]	Y	Y	Y	Y	Y	CT	CT	Y	Y	Y
Far Saeed et al. [10]	Y	Y	Y	Y	Y	CT	CT	Y	Y	Y
Jones et al. [11]	Y	Y	Y	Y	Y	CT	Y	Y	Y	Y
Weldon et al. [12]	Y	Y	Y	Y	Y	CT	Y	Y	Y	Y
Weldon et al. [13]	Y	Y	Y	Y	Y	CT	Y	Y	Y	Y

Y: Yes, CT: Can’t tell.

### Muscular Strength and Power

The frequency and duration of strength and power sessions (offseason and in-season), use of periodization strategies, and prescription of Olympic weightlifting exercises (including derivatives) among S&C coaches are presented in Table 3. Methods used to determine set loads were not included in this review, due to 10 out of 11 methods being inconsistently reported across studies. One consistently reported method was the use of a percentage of repetition maximum tests, which was included in this review, and the percentage of S&C coaches using this method is presented in Table 3. The most important resistance exercise programmed by S&C coaches in all studies was the squat (including variations) (e.g., back squat, front squat, and overhead squat) [6–13]. The second most important resistance exercise was Olympic weightlifting (including derivatives) in five studies (e.g., hang clean, power clean) [6–7, 9–11], deadlift (including variations) in two studies [12–13], and lunge (including variations) in the remaining study [8]. The third most important resistance exercise was the bench press (including variations) in two studies [6, 11], lunges (including variations) in two studies [7, 12], and lat pull-down [8], leg press [10], and Olympic weightlifting (including derivatives) [13] in the remaining studies.

**TABLE 3:** Comparison of results from surveys investigating the practices of strength and conditioning coaches in professional sports.

References				Ebben et al. [6]	Ebben et al. [7]	Ebben et al. [8]	Simenz et al. [9]	Far Saeed et al. [10]	Jones et al. [11]	Weldon et al. [12]	Weldon et al. [13]	Mean
Sample Size				26	23	21	20	100	43	52	33	40

**Strength and Power**	Percentage of coaches who use periodization strategies to structure programs.			69%	91%	86%	85%	100%	88%	98%	97%	89%

	Number of strength/power development sessions delivered per week (p.wk) during the off-season.	1 p.wk		4%	4%	0%	5%	0%	0%	8%	0%	3%
		2 p.wk		4%	0%	0%	0%	22%	7%	44%	27%	13%
		3 p.wk		27%	22%	24%	50%	70%	26%	62%	64%	43%
		4 p.wk		73%	52%	71%	70%	0%	58%	27%	30%	48%
		5 p.wk		8%	26%	5%	25%	0%	23%	12%	9%	13%
		6 p.wk		0%	0%	0%	0%	0%	9%	4%	9%	3%

	Number of strength/power development sessions delivered per week (p.wk) during the in-season.	1 p.wk		4%	n/a	0%	0%	n/a	2%	35%	52%	15%
		2 p.wk		46%	n/a	62%	70%	n/a	33%	62%	67%	57%
		3 p.wk		46%	n/a	29%	65%	n/a	81%	40%	42%	51%
		4 p.wk		27%	n/a	10%	20%	n/a	9%	15%	12%	16%
		5 p.wk		0%	n/a	5%	10%	n/a	2%	4%	0%	4%

	Duration (minutes) of strength/power development sessions delivered per week during the off-season.	0–15 m		n/a	0%	0%	0%	12%	0%	4%	0%	2%
		16–30 m		n/a	0%	0%	5%	14%	5%	17%	3%	6%
		31–45 m		n/a	13%	24%	20%	23%	9%	54%	30%	25%
		46–60 m		n/a	78%	76%	40%	42%	51%	37%	57%	54%
		> 60 m		n/a	9%	0%	40%	9%	30%	27%	54%	24%

	Duration (minutes) of strength/power development sessions delivered per week during the in-season.	0–15 m		0%	0%	10%	n/a	n/a	n/a	4%	3%	3%
		16–30 m		15%	0%	76%	n/a	24%	5%	42%	46%	30%
		31–45 m		58%	13%	14%	n/a	27%	28%	58%	70%	38%
		46–60 m		15%	78%	0%	n/a	47%	60%	30%	42%	39%
		> 60 m		12%	9%	0%	n/a	2%	16%	10%	15%	9%

	Percentage of coaches who prescribe Olympic weightlifting exercises.			88%	91%	29%	95%	83%	88%	67%	88%	79%

	Percentage of coaches who use percentage of repetition maximum to determine set loads.			42%	35%	19%	30%	37%	72%	29%	33%	37%

**Speed**	Exercises used for speed development.	Speed		n/a	83%	n/a	100%	78%	58%	83%	88%	82%
		Plyometrics		65%	83%	81%	90%	92%	30%	87%	88%	77%
		Form Running		77%	43%	100%	80%	69%	30%	38%	64%	63%
		Resisted Running		65%	65%	52%	70%	24%	30%	52%	55%	52%
		Speed Endurance		81%	78%	86%	90%	33%	0%	33%	45%	56%
		Over-Speed Running		58%	43%	19%	35%	71%	0%	19%	15%	33%

**Plyometrics**	Exercises used for plyometrics.	Multiple Hops/Jumps/Lunges		65%	78%	67%	85%	68%	74%	89%	85%	76%
		Box Drills		58%	91%	71%	85%	61%	74%	79%	73%	74%
		Jumps in Place		46%	83%	86%	80%	82%	74%	54%	79%	73%
		Bounding		65%	70%	62%	80%	n/a	72%	79%	79%	72%
		Upper Body		46%	87%	76%	100%	65%	63%	12%	48%	62%
		Standing Jump		46%	39%	33%	50%	74%	70%	63%	82%	57%
		Depth Jumps		27%	22%	10%	40%	66%	63%	56%	67%	44%

	Purpose for using plyometric exercises.	Speed Development		62%	70%	71%	80%	80%	n/a	81%	75%	74%
		Lower Body Power		50%	74%	81%	90%	54%	37%	87%	70%	68%
		Upper Body Power		42%	70%	48%	85%	64%	n/a	2%	36%	50%
		Improve Jumps		n/a	n/a	10%	90%	34%	n/a	63%	36%	47%
		Total Body Training		46%	70%	52%	85%	31%	n/a	8%	30%	46%

	Stage of season that coaches use plyometric exercises.	Year Round		15%	57%	38%	45%	64%	56%	71%	73%	52%
		Pre-Season		27%	35%	n/a	40%	48%	42%	23%	24%	34%
		Pre-Training Camp		19%	26%	43%	40%	52%	12%	n/a	n/a	32%
		In-Season		12%	30%	33%	15%	16%	47%	25%	15%	24%
		Training Camp		4%	30%	33%	10%	40%	5%	4%	6%	17%
		Post Season/Off-Season		23%	22%	10%	35%	16%	1%	2%	9%	15%

	Methods used for integrating plyometric exercises within training programs.		Complex Training/Within Weight Training	27%	57%	43%	60%	14%	58%	52%	52%	45%
			Before Weights	35%	39%	48%	45%	16%	0%	37%	55%	33%
			Separate Days	15%	43%	29%	45%	35%	0%	33%	42%	30%
			After Weight Training	23%	17%	10%	10%	35%	0%	27%	9%	16%

**Flexibility**	Types of flexibility exercises used.	Static		85%	87%	100%	100%	93%	70%	92%	91%	90%
		Dynamic		54%	61%	81%	90%	30%	86%	96%	100%	75%
		PNF		69%	65%	71%	75%	13%	60%	68%	88%	64%
		Ballistic		31%	17%	19%	25%	n/a	n/a	69%	61%	37%

	When coaches prescribe flexibility exercises.	Before Practice		92%	78%	95%	90%	72%	79%	79%	76%	83%
		After Practice		58%	83%	62%	65%	70%	63%	58%	38%	62%
		Before Workout		69%	52%	67%	65%	72%	72%	31%	42%	59%
		After Workout		54%	70%	71%	65%	69%	58%	40%	33%	58%
		Independently		42%	43%	48%	30%	39%	60%	54%	48%	46%
		During Workout		8%	17%	19%	30%	2%	37%	8%	10%	16%
		During Practice		15%	39%	10%	25%	2%	9%	10%	10%	15%

	Duration (minutes) of flexibility sessions.	0–5 m		4%	13%	5%	0%	10%	7%	33%	30%	13%
		6–10 m		46%	26%	14%	15%	43%	35%	67%	70%	40%
		11–15 m		42%	22%	52%	40%	36%	23%	46%	55%	39%
		16–20 m		4%	17%	24%	25%	7%	16%	25%	27%	18%
		> 20 m		0%	4%	5%	15%	4%	9%	10%	15%	8%

**Physical Testing**	Types of physical tests used.	Body Composition		77%	87%	100%	95%	50%	93%	87%	97%	86%
		Muscular Strength		50%	100%	33%	75%	97%	81%	81%	82%	75%
		Muscular Power		35%	83%	33%	85%	97%	86%	62%	76%	70%
		Cardiovascular Endurance		42%	78%	24%	60%	52%	81%	92%	97%	66%
		Speed		35%	7%	19%	80%	100%	86%	81%	91%	62%
		Flexibility		31%	70%	33%	75%	86%	63%	48%	52%	57%
		Anaerobic Capacity		35%	83%	43%	50%	91%	72%	31%	36%	55%
		Acceleration		77%	35%	5%	4%	67%	81%	56%	67%	49%
		Agility		35%	30%	33%	70%	71%	33%	40%	61%	47%
		Muscular Endurance		19%	70%	5%	50%	97%	40%	8%	61%	44%
		Anthropometry		19%	35%	14%	60%	38%	n/a	63%	76%	44%

	Stage of season when physical testing is conducted.	Pre-season		42%	70%	81%	75%	75%	95%	58%	33%	66%
		In-season		31%	52%	62%	60%	46%	88%	42%	24%	51%
		Post/Off-Season		23%	30%	33%	70%	32%	53%	4%	27%	34%

n/a: Data was not attainable from the results of the study. PNF: Proprioceptive neuromuscular facilitation

### Speed

In six studies, 100% of S&C coaches used speed development exercises [6, 8–10, 12–13], with 96% [7] and 93% [11], in the other two studies. The main exercises used for speed development and the percentage of S&C coaches prescribing each exercise are presented in Table 3.

### Plyometrics

In four studies, 100% of S&C coaches used plyometric exercises [9–10, 12–13], 95% in two studies [8, 11], and 91% [7] and 73% [6] in the remaining two studies. Table 3 presents the type of exercises, purpose, method of integration, and period of the season that S&C coaches usually prescribe plyometric exercises.

### Flexibility

In five studies, 100% of S&C coaches used flexibility development exercises [6, 8–9, 12–13], with 96% [7], 95% [11] and 86% [10] in the remaining three studies. Table 3 presents the type of exercises, time of integration, and duration of sessions that S&C coaches use to prescribe flexibility exercises.

### Physical Testing

In six studies, 100% of S&C coaches used physical testing with their athletes [7–10, 12–13], with 98% [11] and 92% [6] in the remaining two studies. The most commonly used physical tests and the time of year where these tests are mostly conducted are presented in Table 3.

## DISCUSSION

The findings of this review revealed that S&C coaches across professional sports mostly apply practices in line with S&C guidelines and research. For some practices differences were observed, suggesting preferential practices, sport-specific exercises, or external constraints may influence S&C programs in certain sports. The reasons and implications of these findings and potential limitations are discussed hereafter.

### Muscular strength and power

Periodization strategies were widely used by S&C coaches across all sports in this review. This is logical given periodization provides the ability to systematically and sequentially integrate training interventions to maximize performance (e.g., strength) at specific time-points (e.g., competition) [21]. But within athletic populations, there is a need for further research to elucidate the usage and long-term effects of periodization, particularly when implementing tapering and unloading strategies [16, 22–23]. In fact, in elite sport relatively little is known regarding the use of periodization, where it is believed that sports and S&C coaches may anecdotally employ periodization strategies based on their philosophies, coaching experience, and available data [16-17]. In this review, American football S&C coaches used periodization the least [6]. Some S&C coaches within this study reported that they started physical training conservatively, and gradually increased the intensity while providing adequate recovery, and challenging athletes when they are fit and healthy [6]. It may be argued that this is a form of periodization, using a less strategic and more dynamic approach. However, there is currently no existing data on the periodization strategies used in professional American football (e.g., National Football League), therefore it is difficult to infer whether such practices are representative of other S&C coaches in this sport [24].

Almost all S&C coaches in soccer [12] and cricket [13] reported using periodization strategies. However, within these studies, S&C coaches also acknowledged that the implementation of periodization strategies was one of their biggest issues, due to limited time, condensed schedule/fixtures, and training volume. Particularly in professional cricket, applying periodization strategies is problematic due to the possibility of players participating for their country, club, and franchise teams, across short and long game formats [13]. Furthermore, with the increased popularity of shorter game formats (i.e., Twenty20), the physical demands players are exposed to in regards to match intensity and number of fixtures played has considerably increased [25]. Therefore, it is encouraging to see the extensive use of periodization strategies in cricket, with the perceived aim of monitoring and manipulating training volume to optimize players’ performance and reduce the likelihood of injuries.

A trend was observed across studies for reducing training volume (e.g., frequency and duration) of strength and power development sessions during the in-season compared to the off-season period. Generally, the competitive or in-season period consists of a combination of peaking and maintenance, which manipulates training loads and volume, to ensure players are in optimal performance for competition and adequate recovery is provided post-competition [21, 26]. Similarly, S&C coaches in rugby union [11], soccer [12], and cricket [13] suggested that training volume should intentionally be reduced during the in-season to adopt a maintenance approach. Furthermore, it has been deemed important by S&C coaches to provide adequate recovery between strength and power development sessions and competition, with 48 hours being the most commonly employed [4, 12–13, 26]. However, outside of competition, strength and power development sessions in soccer [12] and cricket [13] were most commonly held on the same day as sports training sessions. This suggests a more condensed nature of training in-season with less time available for S&C, which is coherent with research recommendations to have a greater emphasis on sport-specific training while in-season [27]. Whereas, during the off-season, S&C coaches can more comprehensively develop the physical capacities of players without negatively impacting sports performance or increasing the likelihood of injury [12, 28].

Various methods were used for determining set loads across reviewed studies, which highlighted inter-and intra-sport differences, suggesting the methods used may be based on the preference of S&C coaches. More recently, studies reporting the practices of S&C coaches have included alternative methods for determining set loads, such as velocity-based training [4, 12], which has also gained increasing popularity in S&C research [29]. Therefore, in this case, it may be considered that contemporary research is potentially driving the diversification of S&C practices. Nevertheless, one consistent method used in all studies was percentages of repetition maximum tests, which is known to be effective in improving strength and power-related capacities in different populations [30]. Furthermore, this method allows S&C coaches to easily prescribe lighter and heavier loads across a week, helping manage athletes’ residual fatigue and preventing over-reaching [30]. This is important in professional sports to ensure athletes remain able to conduct sport-specific training at the required intensity [30]. Across the reviewed sports, S&C coaches in baseball reported testing strength, power, and using percentages of repetition maximum tests the least [8]. This is unexpected given the importance of prescribing adequate training loads to effectively develop strength and power, and the underpinning importance of these physical attributes for batting and pitching performance [31–32]. However, the extensive demands of a long season and intensive playing schedule may limit the time available for testing [8, 33].

The most important resistance training exercise prescribed across sports was the squat (including variations) (e.g., back squat, front squat, and overhead squat) [6–13]. This is unsurprising given the squat provides numerous benefits, including the development of lower body strength and power [34], which can transfer positively to athletic performance (e.g., sprinting) [35]. For example, in American football athletes, one-repetition maximum back squat strength was moderately correlated with sprinting capabilities over 0–5 yds (r = -0.45), 0–10 yds (r = -0.54), and 0–40 yds (r = -60) [36], and strongly correlated in elite soccer athletes over 0–10 m (r = 0.94) and 0–30 m (r = 0.71) [37]. Additionally, back squat performance in conjunction with a wider battery of tests (e.g., squat jump) has been suggested as an indirect measure and reliable predictor (r = 0.75) of sprint performance in rugby union athletes over 0–30 m [38]. Research suggests that variations of the barbell back squat, such as the safety bar back squat provide similar improvements in lower body strength, power, and sprinting performance in baseball players, with the additional benefit of reducing stress on the shoulders and elbow joints [39]. Whereas, in wrestling it has been proposed that back squat strength may also be used to differentiate athletes between levels, with elite wrestlers demonstrating 8–25% greater one-repetition maximum squat values compared to sub-elite wrestlers across weight categories [40]. Therefore, given the overwhelming evidence of the benefits of the squat (including variations), it is logical that S&C coaches extensively prescribe this exercise within their programs.

The second most important exercise reported was Olympic weightlifting (including derivatives) in five out of eight sports [6–7, 9–11]. Olympic weightlifting is widely used to provide a stimulus that effectively trains the whole body, and importantly emphasizes triple extension, a key movement pattern in many sporting actions [41]. Research suggests integrating Olympic weightlifting within an S&C program can improve jumping, sprinting, and change of direction performance [41–43]. However, in baseball, although the aforementioned attributes are required, few S&C coaches used Olympic weightlifting or derivative lifts within their programs [8]. Whereas, S&C coaches seemed to use alternative and highly-specific exercises, such as plyometrics to develop jumping and form running for sprinting [8]. A purported reason for the lack of integration of Olympic weightlifting in baseball is due to the possibility of injuring the shoulders and wrists [44], but this may not be the case when effectively and progressively coached. Consequently, a feasible explanation is that Olympic weightlifting movements can take time to teach and learn [45], and time availability is scarce in baseball given its long and intensive season [8, 33]. Therefore, it is suggested that S&C coaches may implement less complex Olympic weightlifting derivative movements, which are quicker and easier to learn and can provide similar improvements across the force-velocity (power) curve [45].

### Speed

Speed training was extensively used across all reviewed studies, with speed-specific and plyometric training being the most common methods to enhance speed capacity. Improving linear, multi-directional, single, and repeated-bout sprint ability is essential in numerous sports. For example, in field sport athletes, the use of traditional sprint training improved 0–5 and 0–10 m sprint performance, power production, and reactive strength [46]. In the same study, a plyometric training strategy similarly improved 0–5 and 0–10 m sprint performance and reactive strength, while also increasing step length [46]. A review of different sprint training methods on sprint performance over different distances suggested that sprint training is more applicable to improve speed performance over specific distances, while plyometric training primarily improves acceleration (i.e., 0–10 m) [47]. Therefore, combining sprinting and plyometric training strategies seems advantageous for S&C coaches to simultaneously develop acceleration and speed qualities in athletes.

However, it was observed within this review that some S&C coaches prescribed training exercises to develop acceleration and speed but did not specifically test athletes to ascertain their rate of improvement. For example, in ice hockey, 83% of S&C coaches used speed and plyometrics training, but only 7% tested speed and 35% tested acceleration. With research demonstrating high correlations between on-ice acceleration and jump height, and on-ice acceleration and overall speed [48–51], it would be suggested S&C coaches in ice hockey frequently assess such physical capabilities. However, from the available data, it was unclear the reasons why there was such a disparity between training and testing.

### Plyometrics

Plyometric training was commonly prescribed in all reviewed sports. The extensive use of plyometrics is expected with its numerous benefits for improving athletic performance, including speed over different distances (0–40 m), muscle strength and power, landing mechanics, and resilience to injury [5, 52–53]. In this review, S&C coaches mostly prescribed a combination of slow stretch-shortening cycle (e.g., box drills and jumps in place) and fast stretch-shortening cycle exercises (e.g., multiple hops, jumps, lunges, and bounding). This suitably prepares athletes for the various demands of their respective sports, such as slow stretch-shortening cycle exercises (> 250 milliseconds) for acceleration and standing jumps, and fast stretch-shortening cycle exercises (< 250 milliseconds) for top-speed sprinting and take-off phase in locomotive jumps [54]. This also aligns with S&C coaches declaring the main purposes of using plyometric exercises were for speed development and lower body power [6–13].

In American football plyometric exercises were prescribed the least, but still widely used by three out of four S&C coaches [6]. In this study, S&C coaches provided further explanations as to why they limited plyometric exercises: *“no skipping, hopping”, “caution reinjury to the athlete”,* and *“fewer depth jumps with the larger guys”* [6]. However, it is recommended that plyometric exercises are not eradicated from a training program but adjusted to the specific needs and demands of the sport and athlete. For example, it is advised that athletes over 100 kg, which American footballers often exceed this weight [55–57], limit high-volume, high-intensity plyometric exercises, and depth jumps > 18 inches high [53]. Results from the reviewed American football study [6] indicated that S&C coaches did just this, with depth jumps programed the least and alternative plyometric exercises such as multiple hops, jumps, lunges, and bounds used more frequently. Research suggests the use of plyometric training in conjunction with a resistance training program in collegiate American football athletes over eight weeks, demonstrated improvements in speed (0–36.6 m) and agility (T-drill) performance [58]. Furthermore, recommended that providing adequate rest and recovery when performing plyometric exercises with American football athletes, will help improve movement quality and exercise performance while reducing the likelihood of fatigue and injury [58].

In addition to prescribing various lower body plyometric exercises, all basketball S&C coaches reported using upper body plyometric training [9], and to a greater extent than other reviewed sports [6–8, 10–13]. Research in basketball has demonstrated that a twice per week in-season complex training program over 10 weeks incorporating the upper body power exercise medicine ball power drop was able to significantly improve upper body power performance, determined via medicine ball throw [59]. Furthermore, that when upper body power training was reduced to one session per week or removed from players’ S&C programs, they were able to maintain upper body power scores for up to 16 weeks [59]. This may suggest that regular basketball practice is sufficient in maintaining upper body power performance, given its explosive characteristics [59]. Other research assessing the effects of a six-week resistance training and upper body plyometric program including medicine ball overhead throw, side throw, and forward chest pass, demonstrated improvements in upper body strength and jumping performance [60]. Nevertheless, there were no significant differences observed between the experimental and control group who conducted basketball training only [60]. Therefore, this also highlights the potential of basketball training only to be sufficient in developing certain physical attributes of basketball players [60]. Nevertheless, the aforementioned studies were conducted with adolescent [59] and recreational [60] athletes, where there is a lack of research in elite basketball athletes on the beneficial effects of upper body plyometric training and the practices used by S&C coaches. This may suggest the benefits of such exercises are anecdotal, based on non-published data, or the preferences of S&C coaches in elite basketball.

The results of this review demonstrated that S&C coaches predominantly prescribed plyometric exercises all year round. Research suggests long-term plyometric training programs over 24 weeks can be effective in improving lower-body power [61]. Furthermore, it is recommended in team sports such as soccer that plyometric exercises are integrated within an annual training program [62]. The post-season/off-season is possibly the least frequent time for prescribing plyometrics across sports, due to this time being used to reduce the volume of training and promote recovery [21]. Whereas, during the pre-season, there should be a gradual increase in plyometric training, which can be observed from the results of this review.

Plyometrics were mainly integrated as complex training, which involves the *“conduction of maximal or high-intensity dynamic exercises before performing a lighter-resistance ballistic movement with similar biomechanical characteristics”* [63–64]. Research suggests complex training provides a time-efficient method to simultaneously develop strength and power over a given training cycle [64–65]. Furthermore, it has been recommended that S&C coaches can make further use of recovery periods during complex training to prescribe complementary mobility exercises for non-affected limbs [64]. With S&C coaches in certain sports such as soccer [12] and cricket [13], stipulating their biggest difficulty faced was a lack of time to prepare and develop the physical attributes of players, the use of complex training seems a viable option to overcome this issue.

### Flexibility

Flexibility exercises were used extensively across all reviewed studies. Conducting mobility exercises during a training session was the least preferred time of implementation, with before and after practice being the most common [6–13]. It has been suggested that performing dynamic (e.g., reproducing a movement pattern) and static stretching before resistance training sessions, may reduce the likelihood of injury and optimize performance [66]. Whereas proprioceptive neuromuscular facilitation (PNF) stretching can lead to decreased performance (e.g., the total number of repetitions completed) [66]. This was similar to the most common forms of stretching used across studies, with dynamic and static being the most frequently used, with PNF and ballistic stretching the least. The duration of flexibility sessions across studies included in this review was relatively short (e.g., between 6–15 min). This likely occurred as dynamic flexibility movements can be easily implemented within warm-ups. For instance, research has suggested warm-up activities including cardiovascular exercises (e.g., running at a moderate pace) followed by 7 min of dynamic stretching demonstrated significantly improved flexibility and lower body power performance compared to no stretching [67]. Therefore, it appears S&C coaches across sports generally adhere to researchinformed guidelines regarding the prescription of stretching exercises.

### Physical Testing

In the reviewed studies S&C coaches regularly used physical testing to assess their athletes’ physical performance, with pre-season the most common period to conduct testing [6–13]. Physically testing athletes during the pre-season is important to evaluate their current training state, ascertain who adhered to off-season training programs, and determine how subsequent training programs can be designed and tailored to meet the physical condition of teams and individual athletes [27, 68–70]. Furthermore, superior pre-season physical test scores related to different sporting demands, have been associated with a decreased risk of injury and illness [71].

Body composition was on average the most reported physical test used across sports in this review. A possible reason for its frequent use is that it can be tested quickly at any time, without causing additional fatigue to the athlete. Whereas, other tests may be difficult to implement given the potential to increase athletes’ training volume, which can be problematic in elite sport given the time constraints and limited opportunities for recovery [12–13]. However, measuring body composition is of high importance as it can impact various fitness components, such as the contribution of increased lean body mass towards strength and power improvements [72]. Furthermore, some sports require athletes to maintain certain levels of body composition. For example, linemen in American football and props in rugby union are required to possess superior bodyweight for additional inertia that makes it more difficult for opponents to move them, and higher body fat percentages which support the absorption of impacts from tackles and collisions [72–73]. Other benefits of monitoring body composition include talent identification and positional selection [74–75], tracking athletes through long-term athlete development [75], and highlighting injury risk factors [76].

Muscular strength was on average the second most reported physical test used. The improvement and monitoring of maximum strength is of vital importance, given its relationship with enhanced force-time characteristics, locomotion, and sport-specific performance, while also improving athletes’ resilience to injury (e.g., during collisions or contacts) [77]. In ice hockey, all S&C coaches tested for muscular strength (Ebben et al, 2014), which aligns with the aforementioned rationale of strength underpinning key components of sports performance. Strength development in ice hockey athletes has demonstrated improvements in rate of force development, acceleration, speed, power, and agility [51]. Furthermore, superior strength levels have been associated with decreased injury risk in ice hockey players, with players sustaining a groin injury in-season, possessing approximately 18% lower hip abduction strength and poor abductor to adductor strength ratios [51]. In American football, only half of S&C coaches tested for strength [6], which is surprising given the importance of strength for playing this sport [78], however one S&C coach reported that all lifts within training sessions are monitored and recorded. This may suggest that testing is not run independently but embedded within training sessions using alternative approaches (e.g., one-repetition maximum predictions).

On average the third most conducted physical test across sports was for muscular power. The development and assessment of power are important to underpin general and sport-specific movements including throwing, striking, jumping, accelerating, sprinting, and changing of direction [79]. Within this review wrestling [10], rugby [11], and basketball [9] S&C coaches reported testing power the most. Research in these respective sports encourage practitioners to assess power given its relationship with sport-specific movements, ability to discriminate between higher and lower level players and to measure the efficacy of S&C programs [80–84]. Furthermore, in these sports, physical testing was predominantly conducted during the pre-season which is important to provide baseline normative data for each player, create a basis for training, and monitor athletes’ physical performance throughout a season [85].

### Practical Recommendations

The following recommendations are made from this review, based on the practices of S&C coaches in different professional sports. Periodization strategies can be used to manipulate training volume during different phases of the season, while set loads can be determined using percentage of repetition maximum tests. Squats (including variations) are deemed the most important exercise used by S&C coaches, irrespective of the specific demands of each sport, therefore S&C coaches are encouraged to prescribe this exercise to physically develop athletes. Whereas, to improve acceleration and speed, a combination of speed and plyometric exercises is recommended. Plyometric exercises can be prescribed all year round and implemented efficiently within programs using complex training. Furthermore, when programing plyometric exercises, S&C coaches should include slow (e.g., box drills) and fast (e.g., bounding) stretch-shortening cycle exercises to prepare athletes for different sporting demands. For the implementation of flexibility exercises, it is beneficial to perform these before other training activities (e.g., during warm-up), and to keep flexibility sessions relatively short (e.g., 6–15 min). Finally, it is recommended that physical testing takes place during the pre-season period, to assess a range of general and sport-specific measures, to support the design of training programs tailored to meet individual athlete and sporting needs.

### Limitations

There are limitations to this review that should be considered when interpreting the findings. Four out of eight studies were conducted in the United States of America, which may skew results concerning the practices of S&C coaches specific to this region and therefore may not apply to all coaching populations. As S&C continues to develop in regards to education, research, and professionalism there is a continued modernization of practices, therefore it must be considered that practices may have evolved from the time point of when each study was published and further cross-sectional and longitudinal research on the practices of S&C coaches is required across sports. The reviewed studies were restricted to those that used a survey design with common questions to investigate the practices of S&C coaches; accordingly, the results are not inclusive of all practices of S&C coaches in all sports and the results may not be generalizable to other sports, given their specific demands. Lastly, small sections of data were non-attainable from the reviewed studies, and not applicable (n/a) has been used to represent this.

## CONCLUSIONS

This systematic review has enabled the identification and quantification of practices used by S&C coaches across different professional sports. In general, findings have revealed the practices of S&C coaches are common across sports and adhere to S&C guidelines and research. However, subtle differences were observed in some sports, with the rate of prescription of some practices differing from that suggested in the literature and presenting some specific peculiarities. These particular differences possibly arise from (1) S&C coaches preferring certain practices, (2) practices were topical at the given time of when the study was conducted, (3) practices are potentially attributable to the geographical region of where the study was conducted, or (4) practices were specific to the demands or limitations of different sports. Nonetheless, this review provides valuable information for S&C coaches in various areas that could support the planning, conduction, and review of training programs. Furthermore, can be used to inform the future direction of general and sport-specific guidelines, professional development provisions, and research on S&C practices.

## Funding/conflict of interest

Authors declare this manuscript received no funding nor has any conflict of interest.
